# Supplementary material for: EnzML: multi-label prediction of enzyme classes using InterPro signatures
Source: BMC Bioinformatics. 2012 Apr 25;13:61. doi: 10.1186/1471-2105-13-61 (PMC3483700; doi:10.1186/1471-2105-13-61)
Supplement: Addtional file 5 — The Java code to format the data files, evaluate and predict. The file enzml_java_code.tar.gz contains the Java code used to format database data to ARFF and XML formats, to execute cross and train-test (jackknife) evaluations and to record evaluation results to database. More information is included in the readme.txt file and the Javadoc files. The code can be used with a MySQL database. To use a different database software, other JDBC drivers might be required. [file 1471-2105-13-61-S5.gz › java_code/enzml2011/doc/uk/ac/ed/inf/csb/taweka/DataSetFactory.html]

DataSetFactory


---


|  |  |  |
| --- | --- | --- |
| **Package** \| **Class** \| **Use** \| **Tree** \| **Deprecated** \| **Index** \| **Help** | |  |
| **PREV CLASS**   **NEXT CLASS** | **FRAMES**    **NO FRAMES**     **All Classes** |  |
| SUMMARY: NESTED \| FIELD \| CONSTR \| METHOD | DETAIL: FIELD \| CONSTR \| METHOD |  |


---


## uk.ac.ed.inf.csb.taweka Class DataSetFactory

```
java.lang.Object
  uk.ac.ed.inf.csb.taweka.DataSetFactory
```

---

``` public class DataSetFactory extends java.lang.Object ```

DataSet generates a collection of Weka instances (rows of the ARFF file),
their attributes (column names) and their attribute values (cell values)
The last attribute is the class attribute.

**Version:**
:   20 Feb 2008

**Author:**
:   Luna De Ferrari luna.deferrari-at-ed.ac.uk

---

| **Field Summary** | |
| --- | --- |
| `static java.lang.String` | `DUMMY_VALUE` |


**Constructor Summary**

`DataSetFactory(java.lang.String dataSetName,
java.lang.String className,
uk.ac.ed.inf.csb.utils.OneToManyMap instanceAttributeMap,
uk.ac.ed.inf.csb.utils.OneToManyMap instanceClassMap)`
  
          Constructor for a data set.


| **Method Summary** | |
| --- | --- |
| `weka.core.Attribute` | `generateClassAttribute()`             Generates the class attribute. |
| `weka.core.Instances` | `generateDataSet()`             Create a set of instances and fill them with data |
| `java.lang.String` | `getArffFormatString()` |
| `weka.core.Attribute` | `getAttributeByName(java.lang.String name)` |
| `weka.core.FastVector` | `getAttributes()` |
| `weka.core.Attribute` | `getClassAttribute()` |
| `weka.core.Instances` | `getDataSet()` |
| `java.lang.String` | `getDataSetName()` |
| `uk.ac.ed.inf.csb.utils.OneToManyMap` | `getInstanceAttributeData()` |
| `uk.ac.ed.inf.csb.utils.OneToManyMap` | `getInstanceClassData()` |
| `java.lang.String` | `getLog()` |
| `boolean` | `isLogged()` |
| `void` | `setDebug(boolean doLogErrors)` |

**Methods inherited from class java.lang.Object**

`equals, getClass, hashCode, notify, notifyAll, toString, wait, wait, wait`

**Field Detail**

### DUMMY\_VALUE

```
public static final java.lang.String DUMMY_VALUE
```

**See Also:**: Constant Field Values


**Constructor Detail**

### DataSetFactory

```
public DataSetFactory(java.lang.String dataSetName,
                      java.lang.String className,
                      uk.ac.ed.inf.csb.utils.OneToManyMap instanceAttributeMap,
                      uk.ac.ed.inf.csb.utils.OneToManyMap instanceClassMap)
```

:   Constructor for a data set. It can be visualised as:
    instances (rows), attributes (columns), class (last column), values (cells)

    **Parameters:**: `dataSetName` - the data relation name: `className` - the class/category of the instance: `instanceAttributeMap` - TODO: `instanceClassMap` - TODO


**Method Detail**

### generateDataSet

```
public weka.core.Instances generateDataSet()
```

:   Create a set of instances and fill them with data

    :   **Returns:**: the data set

---


### generateClassAttribute

```
public weka.core.Attribute generateClassAttribute()
```

:   Generates the class attribute.
    Adds a dummy start value (to work around a known weka bug
    described at http://weka.wikispaces.com/ARFF+%28stable+version%29)
    or http://www.cs.waikato.ac.nz/~ml/weka/arff.html)
    From Weka documentation: "Warning: There is a known problem saving SparseInstance objects from datasets that have string attributes.
    In Weka, string and nominal data values are stored as numbers; these numbers act as indexes into an array of possible attribute values (this is very efficient).
    However, the first string value is assigned index 0: this means that, internally, this value is stored as a 0.
    When a SparseInstance is written, string instances with internal value 0 are not output, so their string value is lost
    (and when the arff file is read again, the default value 0 is the index of a different string value, so the attribute value appears to change).
    To get around this problem, add a dummy string value at index 0 that is never used
    whenever you declare string attributes that are likely to be used
    in SparseInstance objects and saved as Sparse ARFF files."

    :   **Returns:**: the class attribute

---


### getArffFormatString

```
public java.lang.String getArffFormatString()
```

---


### getDataSetName

```
public java.lang.String getDataSetName()
```

---


### getAttributes

```
public weka.core.FastVector getAttributes()
```

---


### getAttributeByName

```
public weka.core.Attribute getAttributeByName(java.lang.String name)
```

---


### getInstanceAttributeData

```
public uk.ac.ed.inf.csb.utils.OneToManyMap getInstanceAttributeData()
```

---


### getClassAttribute

```
public weka.core.Attribute getClassAttribute()
```

---


### getDataSet

```
public weka.core.Instances getDataSet()
```

---


### getInstanceClassData

```
public uk.ac.ed.inf.csb.utils.OneToManyMap getInstanceClassData()
```

---


### isLogged

```
public boolean isLogged()
```

---


### setDebug

```
public void setDebug(boolean doLogErrors)
```

---


### getLog

```
public java.lang.String getLog()
```


---


|  |  |  |
| --- | --- | --- |
| **Package** \| **Class** \| **Use** \| **Tree** \| **Deprecated** \| **Index** \| **Help** | |  |
| **PREV CLASS**   **NEXT CLASS** | **FRAMES**    **NO FRAMES**     **All Classes** |  |
| SUMMARY: NESTED \| FIELD \| CONSTR \| METHOD | DETAIL: FIELD \| CONSTR \| METHOD |  |


---
